# A systematic review of ambulance service-based randomised controlled trials in stroke

**DOI:** 10.1007/s10072-023-06910-w

**Published:** 2023-07-05

**Authors:** Mark Dixon, Jason P. Appleton, A. Niroshan Siriwardena, Julia Williams, Philip M. Bath

**Affiliations:** 1 Stroke Trials Unit, Mental Health & Clinical Neuroscience, University of Nottingham, Queens Medical Centre Campus, Nottingham, NG7 2UH UK; 2 East Midlands Ambulance Service NHS Trust, Nottingham, UK; 3 Stroke, Nottingham University Hospitals NHS Trust, Nottingham, UK; 4 School of Health and Social Care, University of Lincoln, Lincoln, UK; 5 Department of Paramedic Science, School of Health and Social Work, University of Hertfordshire, Hatfield, UK

**Keywords:** Systematic review, Stroke, Paramedic, Ambulance, Emergency medical services

## Abstract

**Background:**

Treatment for stroke is time-dependent, and ambulance services play a vital role in the early recognition, assessment and transportation of stroke patients. Innovations which begin in ambulance services to expedite delivery of treatments for stroke are developing. However, research delivery in ambulance services is novel, developing and not fully understood.

**Aims:**

To synthesise literature encompassing ambulance service-based randomised controlled interventions for acute stroke with consideration to the characteristics of the type of intervention, consent modality, time intervals and issues unique to research delivery in ambulance services.

**Summary of review:**

Online searches of MEDLINE, EMBASE, Web of Science, CENTRAL and WHO IRCTP databases and hand searches identified 15 eligible studies from 538. Articles were heterogeneous in nature and meta-analysis was partially available as 13 studies reported key time intervals, but terminology varied. Randomised interventions were evident across all points of contact with ambulance services: identification of stroke during the call for help, higher dispatch priority assigned to stroke, on-scene assessment and clinical interventions, direct referral to comprehensive stroke centres and definitive care delivery at scene. Consent methods ranged between informed patient, waiver and proxy modalities with country-specific variation. Challenges unique to the prehospital setting comprise the geographical distribution of ambulance resources, low recruitment rates, prolonged recruitment phases, management of investigational medicinal product and incomplete datasets.

**Conclusion:**

Research opportunities exist across all points of contact between stroke patients and ambulance services, but randomisation and consent remain novel. Early collaboration and engagement between trialists and ambulance services will alleviate some of the complexities reported.

**Registration number:**

PROSPERO 2018CRD42018075803

**Supplementary Information:**

The online version contains supplementary material available at 10.1007/s10072-023-06910-w.

## Introduction

Stroke is the second-largest cause of death worldwide with an estimated 6.7 million deaths every year and the third-largest cause of disability [1–4]. Ischaemic stroke is a time-sensitive disease, and outcomes are strongly associated with time from symptom onset to definitive treatment with alteplase, with the best outcomes occurring within the golden hour from ictus [5–7].

Treating large numbers of patients in this hyperacute time window requires streamlined care pathways which are difficult to achieve through hyperacute hospital services alone [8]. Considering the time is lost brain concept [9], the role of ambulance services has shifted to ensure early recognition of suspected stroke, undertake prompt assessment at scene and commence rapid transport to the nearest hyperacute stroke centre with prenotification, thus ensuring ischaemic stroke patients benefit from time-sensitive thrombolytic therapy [10–12].

There is increasing recognition that prehospital research is fundamental to further enhance hyperacute stroke care [13]. Interventions to streamline systems of prehospital stroke care from enhanced assessment by telephone and interventions applied at scene or delivered in-transit are all evident within the literature with varying success. Yet, none have been widely adopted as part of the guidelines for prehospital stroke. Despite recent growth of the paramedic profession in the United Kingdom (UK), evidence to understand research approaches, conduct and complexities of research delivery within emergency ambulance service settings is scarce [14–16].

This review seeks to systematically understand the breadth of randomised interventions trialled for ultra-acute stroke within ambulance service settings and to explore the unique characteristics reported when undertaking randomised controlled trials within ambulance services for hyperacute stroke.

## Materials and methods

This systematic review was conducted in accordance with the Preferred Reporting Items for Systematic Reviews and Meta-analyses (PRISMA) guidelines [17] and is registered with PROSPERO (2018CRD42018075803). MEDLINE, EMBASE, Web of Science, CENTRAL and WHO IRCTP databases were searched for randomised controlled trials in hyperacute stroke where an ambulance service-based intervention was tested. No country, language or time limitations were set, and pilot randomised controlled studies were included to fully inform the review. The search was conducted on 10th September 2021, and re-performed on 16th April 2022, to capture recent research. The search strategy can be viewed in the supplementary content.

### Screening

Titles and abstracts of all returned studies were screened by one author (MD), and those meeting the selection criteria were retrieved. Two authors (MD and JPA) independently screened the full papers to confirm the eligibility for inclusion, and any discrepancies were discussed. A record of exclusions was maintained as well as information to support inclusion decisions. The reference lists of included studies were hand-examined for other relevant articles.

A trial was considered randomised if the method of randomisation was clearly defined, or if the trial was reported as randomised, and no evidence of a non-randomised method of allocation was recorded. Eligible studies were included where adult patients presented to ambulance services with symptoms of acute stroke, and the randomised intervention was commenced prior to arrival at hospital.

Papers were excluded that were not original research, not ambulance service-based, involved inter-hospital transfers where patients had received a diagnosis of acute stroke, or comprised a protocol, review or guideline paper.

Risk of bias was assessed in accordance with the Cochrane collaboration’s tool for assessing bias in randomised trials [18].

### Data synthesis

The following data was extracted and recorded in data tables: study design, setting, study period, inclusion criteria, randomisation strategy, intervention and control, primary outcome, sample size, population demographics, type of consent obtained, presence of doctor (none, remote or in ambulance), final diagnosis, timings and logistics and any narrative characteristics or challenges unique to the prehospital research environment. Meta-analysis would be considered if sufficient numerical data allows.

## Results

The search returned 538 references across five databases (MEDLINE, EMBASE, Web of Science, CENTRAL, WHO IRCTP). Thirty articles were identified through the initial title, and abstract screening of which 15 met the full inclusion criteria (Table 1 and Supplement Table 1). Excluded trials are listed in Supplement Table 2. The Cochrane Risk of Bias tool was used to appraise the methodological quality of the studies (Supplement Table 3).

**Table 1:** Summary of trial characteristics

Study	Year	Location	N	Design	Intervention	Control	Inclusion/exclusion	Stroke assessment tool	Primary outcome
De Luca [19]	2009	Italy	4895	Cluster RCT	Prehospital pathway	Standard care	Inc: age <80 <6 hours from symptom onset	CPSS	Proportion of patients referred to stroke unit
Nurmi [20]	2011	Finland	61	Prospective, randomised intervention	Insulin IV or insulin SC	Non-randomised standard care	Inc: plasma glucose >6.0 mmol/l, age between 18 and 80, capacity to provide consent (or next of Kin) on-scene, prior independence <2 hours from symptom onset Exc: malignancy, pregnancy, recent invasive surgery, traumatic injury, persistent hypertension, sever liver disease, alcohol abuse	CPSS	Change in plasma glucose concentration from scene to admission
Berglund [21]	2012	Sweden	942	Randomised controlled trial	Higher priority dispatch code	Normal dispatch disposition	Inc: age between 18 and 85, prior independence <6 hours from symptom onset	FAST	(1) Unproportional interference with other ambulance transports (2) Increase in patients arriving at stroke unit within 6 hours from symptom onset (3) Rate of thrombolysis
MSU [22]	2012	Germany	100	Randomised single centre-controlled trial	Mobile stroke unit	Standard care	Inc: age between 18 and 80, one or more stroke symptoms. <2.hours from symptom onset Exc: uncertain onset, pregnancy	Modified ROSIER	Time from alarm to therapy decision
Hougaard [23]	2013	Denmark	443	Single centre, open-label, outcome observer blinded randomised study	Perconditioning	Standard care	Inc: >18, new hemiparesis and/or aphasia, conscious, prior independence, Teleconference with senior neurologist to confirm eligibility for rtPA. MRI showing lesion consistent with AIS for full inclusion. <4.5 hours from symptom onset	Modified FAST	Penumbral salvage
RIGHT Pilot [24]	2013	Nottingham, UK	41	Paramedic-delivered, ambulance-based single-city prospective single-blind RCT	GTN transdermal patch	Sham patch	Inc: FAST 2 or 3, SBP>140 mmHg, age >40 male, age >55 female <4 hours from symptom onset Exc: BM<2.5mmol/l, GCS <=8, non-ambulatory	FAST	Reduction of systolic BP at 2 hours.
PIL-FAST Pilot [25]	2014	UK	14	Double-blind pilot RCT	Lisinopril	Placebo	Inc: new unilateral arm weakness, within 3 hours of symptom onset, BP >160mmHg, must attend one of 3 participating hospitals <3 hours from symptom onset Exc: contraindications to lisinopril and conditions compatible with stroke mimics	No tool applied	Prehospital enrolment of 4 patients per month
PHANTOM-S [26]	2014	Germany	6182	Randomised week, open-label clinical trial.	Thrombolysis	Standard care	Inc: age >18, calls between 07:00 and 23:00 hours <4 hours from symptom onset Exc: age <18, pregnancy	No tool applied	Alarm-to-needle time
Malekzadeh [27]	2015	Mashad, Iran	246	Quasi-empirical	CPSS telephone triage	Standard care	Inc: age >18, call made by patient or caregiver Exc: life threatening conditions requiring alternative intervention	CPSS	Final diagnosis of stroke
FAST-Mag [28]	2015	USA	1700	3-way multicentre randomised double blind, placebo controlled, pivotal RCT	Magnesium	Standard care	Inc: age >40 and <95, suspected stroke with mLAPSS <2 hours from symptom onset	Modified LAPSS	mRS day-90
RIGHT-2 [29]	2019	UK	1149	Paramedic-delivered, multicentre, randomised controlled trial	Glyceryl trinitrate	Standard care	Inc: FAST 2 or 3, Systolic BP>120 mmHg <4 hours from symptom onset Exc: blood glucose <2.5 mmol/l, GCS <=8, non-ambulatory, resides in nursing home, unable to gain informed consent or proxy assent	FAST	mRS day-90
Larsson [30]	2019	Sweden	19	Randomised controlled trial	Exenatide	Standard care	Inc: age >18, FAST >1, Blood glucose 8-15mmol/1, written informed consent <6 hours from symptom onset Exc: T1DM, antidiabetic treatment (excluding metformin), pregnancy, liver disease, dialysis, GCS <14 of V <5, pharyngeal palsy, dementia	FAST	(1) Reduction of plasma glucose with 2.0 mmol/l 4 hours after randomisation (2) Feasibility of prehospital treatment
Helwig [31]	2019	Germany	116	Randomised multicentre	Optimised prehospital management	MSU	Inc: age>18, cl-FAST >1, wake-up stroke, witnessed informed consent by patient or relative <8 hours from symptom onset Exc: renal dysfunction, pregnancy, allergy to contrast agents, terminal disease, unstable cardiopulmonary conditions	Cl-FAST LAMS	Accurate triage to comprehensive or primary stroke centre
PASTA [32]	2020	UK	1214	Pragmatic, multicentre, cluster RCT	Acute stroke management pathway	Standard care	Inc: FAST >1, age >18 <4 hours from symptom onset PASTA or Standard Care Paramedic transferring to a participating centre	FAST	Proportion of patients receiving thrombolysis
BEST-MSU [33]	2021	USA	1515	Pseudorandomised—alternating week, cluster-controlled trial of a mobile stroke unit	MSU	EMS	Inc: examination features consistent with acute stroke, <4.5 hours since last known well, no obvious contraindications to t-PA	No tool applied—examination features consistent with stroke	mRS at day-90 of 0 or 1

*AIS* acute ischaemic stroke, *BP* blood pressure, *Cl-FAST* Consciousness leg Face Arm Speech Time test, *CPSS* Cincinnati Prehospital Stroke Scale, *CT* computerised tomography, *EMS* Emergency Medical Services, *FAST* Face-Arm-Speech-Time test, *GCS* Glasgow coma scale, *ICH* intra cerebral haemorrhage, *IV* intravenous, *LAMS *Los Angeles Motor Scale, *mLAPSS* modified Los Angeles Prehospital Stroke Screen, *MRI* magnetic resonance imaging, *mRS* modified Rankin Scale, *MSU* mobile stroke unit, *RCT* randomised controlled trial, *ROSIER* Recognition of stroke in the emergency room, *SC* subcutaneous, *T1DM* type 1 diabetes mellitus, *t-PA* tissue plasminogen activator

**Fig. 1:**
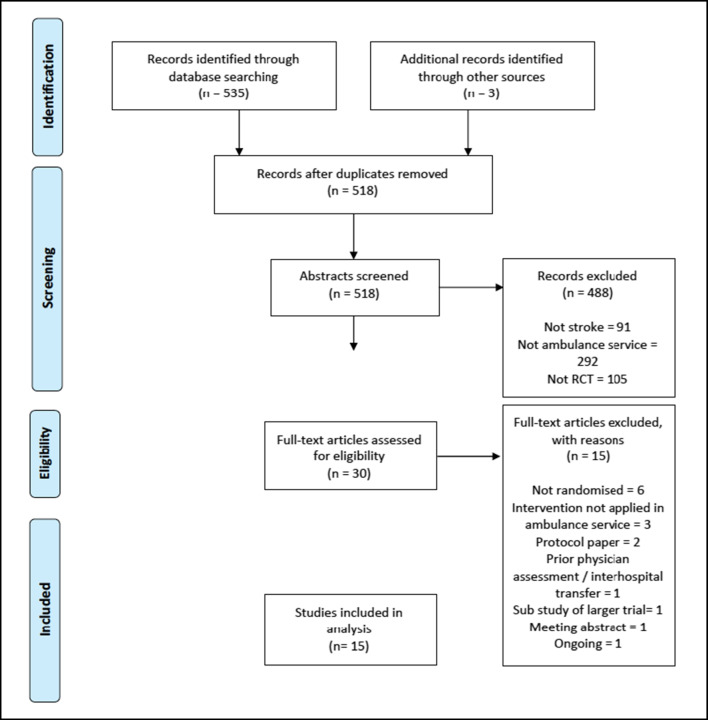
PRISMA flow-chart

Due to the heterogeneity of the designs, population groups, interventions, outcomes, varying comprehensiveness and inconsistency in time intervals reported, it was only partially feasible to undertake a meta-analysis. Mainly, the data lent themselves to a narrative synthesis which was summarised into the headings: randomised interventions tested, consent, time intervals and considerations unique to the prehospital field. Fig. 1

### Study characteristics and interventions

The 15 studies included 18,637 participants and were from Iran [27], the USA [28, 33] and Europe [19–26, 29–32], of which four were in the UK [24, 25, 29, 32]. Interventions were evident across all points of contact with ambulance services. In the control room, a call-handler stroke recognition tool was applied [27]. And a higher dispatch priority was assigned to likely stroke [21]. As part of a larger pathway, an amendment was made to enhance stroke recognition by ambulance dispatchers in the control room where ambulance staff on-scene then conveyed these patients to specialist stroke centres [19].

Clinical interventions were delivered in seven trials of which three administered antihypertensives (paramedic-initiated lisinopril for acute stroke treatment, PIL-FAST [25], rapid intervention with glyceryl trinitrate in hypertensive stroke trials: RIGHT [24], RIGHT-2 [29]), one a neuroprotectant (field administration of stroke therapy-magnesium, FAST-MAG [28]), two glucose control [20, 30] and one tested mechanical ischaemic per-conditioning [23].

One trial explored enhanced on-scene assessment by paramedic and expedited transfer to comprehensive stroke centres (CSC) (paramedic acute stroke treatment assessment, PASTA [32]). Lastly, four trials investigated mobile stroke unit (MSU) care comprising computerised tomography (CT) imaging to confirm stroke diagnosis, deliver thrombolysis at scene or in-transit to assess timeliness of delivery and level of disability versus standard care (benefits of stroke treatment delivered by mobile stroke unit compared with standard management by emergency medical services, BEST-MSU [33], diagnosis and treatment of patients with stroke in a mobile stroke unit, MSU [22], Helwig et al*.* [31], and prehospital acute neurological treatment and optimisation of medical care in stroke, PHANTOM-S [26]).

Primary outcomes can be divided into four distinct groups of, triage/referral decisions and diagnosis [19, 21, 27, 31, 32], time influence [22, 26], physiological parameters (including mRS) [20, 23, 24, 28–30, 33] and feasibility of prehospital intervention [25]. This suggests interest is high with recognition to the potential influence of prehospital intervention for stroke, yet safety, feasibility and impact remain largely unexplored.

### Summary of randomisation

A range of randomisation methods were applied. Malekzadeh et al., a control room-based study, quasi-randomly assigned calls as they were received to triage nurses who were either trained or not trained in the trial process [27]. Four trials relied on envelope randomisation: Berglund et al. in the control room determined the assignment of a normal or higher priority ambulance response by envelope [21], and two on-scene intervention trials used sealed sequenced envelopes stored on ambulance vehicles [20, 24]. The study by Hougaard et al. [23] relied upon envelope randomisation managed off-site by an on-call staff nurse or physician who received a telephone call from a clinician at the scene. Four drug intervention trials allocated randomised treatment packs to ambulances or packs were individually issued to paramedics [24, 25, 29, 30]. A cluster-randomised design due to large geographic areas with multiple participating sites and hospitals was appropriate in De Luca et al*. *[19] and paramedic acute stroke treatment assessment (PASTA), [32] where PASTA randomised paramedics themselves reducing the complexity of additional randomisation activity at the emergency scene [32]. Randomised/alternating week schedules were applied in four studies bringing mobile stroke units to the scene due to the size of the trial and complexity of the intervention [22, 26, 31, 33]. Randomisation bias was addressed in the randomisation schedule of six studies [22, 25, 26, 28, 29, 33].

### Consent

All studies reported consent mechanisms with twelve actively seeking informed consent that was either verbal or full (Table 2).

**Table 2:** Consent methods by study

Study	*N* (%)	Patient	Proxy–relative/friend/carer	Proxy–paramedic	Doctor (at scene)	Doctor (remote/at hospital)	Comments
De Luca [19]	4895	*-*	*-*	*-*	*-*	*-*	Consent not required–service change only
Nurmi [20]	61			*-*	*-*	*-*	Patient/next of kin informed consent. N not disclosed
Berglund [21]	942	*-*	*-*	*-*	*-*	*-*	Consent not required–service change only
Malekzadeh [27]	246	-	-	-	-	-	Control room nurses provided consent, waived from patient consent due to time-critical circumstances
MSU [22]	100	-	-	-	53 (53)	47 (47)	Patient or legal representative consent taken by doctor
Hougaard [23]	443	-	-	-	-	443 (100)	Provisional consent by Paramedics. Full consent sought in hospital from patient or proxy. Authors report unknown number of patients in control group lost to follow up as consent not completed
PIL-FAST [25]	14	10 (71)	1 (7)	3 (21)	-	-	Verbal consent in ambulance, written consent followed up at hospital. One withdrawal due to relative distress, unable to gain continued consent
RIGHT [24]	41	9 (22)	20 (49)	12 (29)	-	-	Patient assessed for capacity where written consent from patient, relative or Paramedic Consent reconfirmed at hospital
PHANTOM-S [26]	6182	*N not reported*					Patient informed consent where patients had capacity or waived consent from those who could not communicate. N not reported
FAST-Mag [28]	1700	-	-	-	-	1700 (100)	Written informed patient consent if competent to provide it, or from legally authorised representatives by telephone with physician: 1017 (60%) patient written consent 662 (39%) legally authorised representatives 21 (1%) exemption from explicit consent
RIGHT-2 [29]	1149	603 (53)	431 (37)	115 (10)	-	-	Patient assessed for capacity where written consent from patient, or when unable to gain informed consent–written assent from relative or Paramedic with colleague as witness
Larsson [30]	19	19 (100)					Written informed patient consent required for inclusion by physician over the telephone
Helwig [31]	116			-	-	-	Patient or relative written consent required; N not specified. Screening prior to enrolment excluded 44 who denied consent
PASTA [32]	1214			-	-	1214 (100)	Written informed consent sought from patient or relative by Doctor after arrival at hospital Breakdown of patient or relative informed consent not provided
BEST-MSU [33]	1515	*N not reported*					Written informed consent was obtained from all patients or their representative, breakdown not reported

Where provisional consent was sought in the ambulance, this was followed by in hospital following positive imaging [32]. Hougaard et al. required the potential participant or their legal representative to undertake a telephone consultation with a remote physician who randomised over the telephone once consent was given [23].

FAST-MAG [28] required remote telephone confirmation of eligibility with a medic who enrolled where appropriate. Larsson et al. [30] had on-call physician availability for consultations throughout the study period, but screening and inclusion were conducted by registered prehospital nurses. Consent by physician was taken in the MSU studies [22, 26, 31, 33].

The control centre-based trials did not take consent from patients. Two implemented service changes only with no change of clinical practice at the scene [19, 21]. The remaining trial consented the telephone triage nurses who were trained to undertake the protocol in the control room but waived patient consent due to the time-critical nature of stroke [27].

The two UK-based pilot trials, PIL-FAST and RIGHT, tested a method of consent where paramedics assessed the capacity of the patient and obtained informed consent where capacity was demonstrated [24, 25]. Where capacity was lacking, proxy consent was sought from a relative or carer on-scene or provided by the paramedic witnessed by a colleague where no representative for the patient was on-scene. This approach was later used in the large follow-on RIGHT-2 study using the same method [29].

One study failed to complete the consent process for patients enrolled in the control group after initial consent had been sought in the ambulance and resulted in a randomisation imbalance. These data were lost to follow up, and the number of patients that were lost is unknown [23].

Nurmi et al., benefits of stroke treatment in an MSU (BEST-MSU), and Berglund et al. did not report a breakdown by source of consent; however, all indicate that patient consent or next of kin informed consent was obtained with the latter waiving consent in line with hospital routine practice in definitive stroke care [20, 26, 33].

### Time intervals

**Fig. 2:**
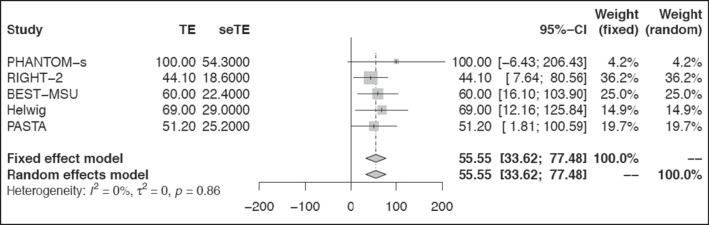
Time interval: call/alarm to treatment forest plot

Thirteen of 15 studies reported time intervals [19–22, 24–29, 31–33]. Variation in terminology and time-interval points reported between studies allowed partial meta-analyses (Supplement Figures 1, 2, 3, 4, and 5). Promisingly, five reported a decrease in time from stroke onset to definitive care despite interventions prolonging the prehospital phase (Supplement Table 4) [20, 21, 28, 29, 33]. Meta-analysis of the call/alarm to treatment indicates ambulance-based intervention can be administered within 55 min (Fig. 2).

**Fig. 3:**
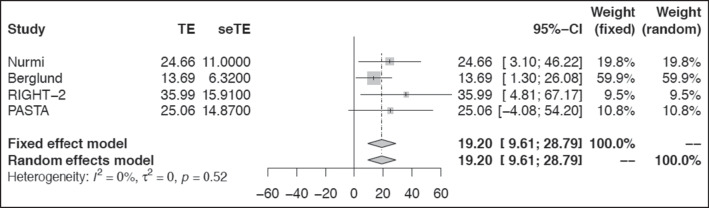
Time interval: total time on-scene forest plot

The field administration of stroke therapy-magnesium (FAST-MAG) and RIGHT-2 trials reported the additional interventions applied on-scene for stroke did not compromise ambulance response timings and were viewed favourably as improving the quality of care offered to stroke patients [28, 29]. Importantly, meta-analysis of total time on-scene from four trials reporting arrival and departure times confirmed that mean timeframes remained low, despite the addition of research activity, in congruence with current practice recommendations in prehospital stroke (Fig. 3) [20, 21, 29, 32]. Three trials administering treatment interventions at scene (FAST-Mag, PIL-FAST, RIGHT-2) were comparable in time from arrival at scene to intervention delivery of 22.8 min (supplement figure 4).

Finally, two studies where ambulances bypassed local hospitals in favour of conveying patients directly to comprehensive stroke centres reported contrasting results. In Sweden, the increased priority afforded to stroke did not adversely affect response to other waiting emergency calls [21]; however, a region within the cluster trial by De Luca et al. withdrew their participation after emergency ambulance resources travelled long distances to the stroke centre and reduced resource availability, prolonging waits for patients in the community [19].

### Unique prehospital considerations

Studies reported numerous characteristics unique to research conducted in ambulance-based settings (Supplement Table 5).

### Presence of physician

The presence of a physician (in ambulance, remote or none; Table 3) to determine the accuracy of the final diagnosis was difficult to assess. Trials where remote physician support to assess eligibility, seek consent and randomise recruited the lowest mimic rate and highest ischaemic stroke inclusion which supports specialist involvement. This decreased as anticipated with no physician presence, but detection rates remain consistent with the literature. MSU control groups were often given standard care without physician presence, so the data must be interpreted with caution.

**Table 3:** Final diagnosis, by the presence of doctor

Physician presence	Study	*N* (%)	IS	ICH	TIA	Mimic
In ambulance	MSU [22]	100	54 (54)	11 (11)	17 (17)	18 (18)
	PHANTOM-S [26]	6182	2111 (34)	145 (2)	643 (10)	3283 (53)
	Helwig [31]	116	71 (61)	16 (14)	21 (18)	8 (7)
	Best-MSU [33]	1515	1047 (69)	218 (14)	56 (4)	190 (13)
	Total	7913	3283 (41)	390 (5)	737 (9)	3422 (44)
Remote	Hougaard [23]	443	240 (54)	37 (8)	58 (13)	105 (24)
	FAST-Mag [28]	1700	1245 (73)	387 (23)	0 (0)	67 (4)
	Total	2143	1485 (70)	424 (20)	56 (3)	172 (8)
None	Nurmi [20]	61	36 (60)	9 (15)	7 (11)	3(5)
	Berglund [21]	942	316 (34)	46 (5)	134 (14)	446 (47)
	RIGHT [24]	41	27 (66)	6 (15)	3 (7)	5 (12)
	PIL-FAST [25]	14	9 (64.3)	3 (21.4)	1 (7.1)	1 (7.1)
	De Luca [19]	4895	ICD9CM group 2971 (61)			1924 (39)
	Malekzadeh [27]	246 (116 recognised as stroke and included)	57 (49)	3 (3)	16 (14)	40 (34)
	RIGHT-2 [29]	1149	597 (52)	145 (13)	109 (9)	297 (26)
	Larsson [30]	19	13 (68)	2 (11)	0 (0)	4 (21)
	PASTA [32]	1214	1016 (83)	196 (16)	0 (0)	1 (<1)
	Total	3556	2071 (58)	407 (11)	270 (8)	803 (23)
	All	13612	6839 (50)	1224 (9)	1065 (8)	4468 (33)

Totals exclude: De Luca due to no differentiation of diagnoses; Malekzadeh reported final diagnosis of patients who were recognised as stroke. RIGHT-2: *n* = 1 stroke type not confirmed. Nurmi: *n* = 6 diagnoses not confirmed. BEST-MSU: *n* = 3 unclear final diagnosis, *n* = 1 confirmed missing. Hougaard: *n* = 3 not included. PASTA enrolment confirmed after arrival at hospital, 1 patient not included as ICH but treated as IS

For ambulance personnel, accuracy of identifying stroke remains a significant challenge due to varied clinical presentations, time pressures on-scene and a reliance on symptom-validated tools which have moderate-to-good sensitivity but lower specificity, such that 30–50% of patients identified as having suspected stroke later receive an alternative non-stroke or mimic diagnosis [34, 35].

### Low recruitment

The pre-planned sample size was not met during the recruitment phase of the three trials [24, 25, 30] with one achieving a rate of one patient per month [25]. Half the planned sample size was achieved in RIGHT [24] and a further trial stopped early due to low recruitment [23]. PIL-FAST investigators reported that screening of paramedic records indicated 6–7 patients were eligible per month, but only 33% were attended by a paramedic trained in the trial of which 54% were then enrolled [25]. Recognising low recruitment as a rate-limiting factor, four trials broadened and modified eligibility criteria, and recruitment phases were extended to overcome this phenomenon [21, 24, 29, 30].

RIGHT-2 increased the number of participating ambulance services from five to eight and stroke centres from 30 to 54 during the recruitment phase. Despite this, 516 of 1492 (36%) paramedics trained in RIGHT-2 trial procedures recruited at least one patient. Furthermore, recruitment hours initially limited to typical working hours for research staff availability were extended in RIGHT-2 to encompass 24/7 recruitment reflective of real-world ambulance care to not limit participation and maximise inclusion [29].

Consequently, re-familiarisation with recruitment protocols throughout the recruitment phase and recruitment of new paramedic or ambulance nurse researchers was necessary as some recruitment phases exceeded 60 months [28, 30, 33]. RIGHT investigators delivered 22 face-to-face training sessions for paramedics yet recruited only 41 patients [24]. De Luca et al. also reported a low rate of patient referral despite being resource-intensive and having collaborative training and a coordination programme [19].

### Data inconsistencies

Three trials reported data collection inconsistencies. First, data information sheets were missing in 55.5% of patients enrolled in to the intervention arm of PASTA despite efforts to encourage completion [32]. Second, inconsistencies in ambulance staff recording of key times relating to symptom onset necessitated a protocol change in the study of De Luca et al. [19]. Third, 38 prehospital protocol violations were recorded in RIGHT-2 [29] highlighting that operational feasibility and data collection is challenging with limited staff and time constraints in the prehospital arena.

### Investigational medicinal product (IMP) management

Two studies (PIL-FAST, RIGHT-2) where IMP was stored and randomised within ambulances experienced mislaid IMP. Crucially, the handover point between ambulance and hospital has been identified as a key risk for loss as both reported losses during the transition of care at the point of clinical handover [25, 29]. Seasonal temperature variance in ambulances was considered in the study of Larsson et al. where steps to mitigate this by stock rotation and storage in insulated cases were applied [30].

### Methodological quality

All studies were successful in their approach to applying interventions in the ambulance service environment. Two achieved partial success, and one study was limited due to the oversight in capturing re-consent of patients in the control arm, rendering data unreportable [23]. The study of Larsson et al. was stopped early as the recruitment rate was low [30], and the De Luca et al. trial withdrew a cluster region from participation as other waiting emergency calls were compromised as ambulance resources were travelling further to reach stroke unit care [19]. PIL-FAST [25] and RIGHT [24] were pilot trials, and the authors of one study had previously undertaken a non-randomised pilot [36] to assess the feasibility for a larger review that was not eligible for inclusion in this paper.

## Discussion

This review is the first to explore the types of randomised intervention and characteristics of ambulance service-based randomised controlled studies in stroke. Randomised interventions were identified across the chain of contact with ambulance services from detection of stroke and dispatch, clinical intervention on-scene and in-transit, direct referral to primary stroke centres and definitive stroke care delivery at the scene. Aligning to the time-is-brain concept [9] all included trials complement global efforts to reduce time within the prehospital phase of care [13, 37–39].

The majority of interventions focused on paramedic-led administration of interventions on-scene or in-transit. These are simple, time-efficient, well-suited to the existing prehospital provider’s skillset and widely distributable across large geographical regions without adding significant workload to what can often be disorganised, chaotic and challenging environments [40–42]. Whilst MSUs clearly provide shorter times to definitive care and improved outcomes when imaging is performed in the field, MSU coverage is still limited by driving distances, times of operation and high cost of implementation [43, 44].

Recruitment rate must be considered a real-world factor in planning and facilitating research in prehospital care, and this has been described elsewhere [45]. From a UK perspective, in a system where response time is one benchmark of the quality of ambulance service provision, ambulance dispatchers are not routinely able to assign specific research-trained personnel to specific emergency calls, instead allocating the nearest available resource to attend. As UK paramedics voluntarily participate in research, records suggest that only one-third of the paramedic workforce participate [16] which further complicates directing a research-trained paramedic to potential participants. From this review, it is clear that recruitment phases are lengthy with a need to re-train staff and often extend geographical coverage from pre-planned settings and increase resource production (e.g. investigational medicinal product), which can impact upon funding.

The method of randomisation offers a potential solution. Cluster randomisation by the ambulance station randomises a group of paramedics themselves without adding steps at the scene. This can allow paramedics to carry out research activity without impacting upon clinical care with fewer stages at the patient’s side [32, 46]. Promisingly, this method could provide a viable solution to the low recruitment seen across trials in this review and avoid lengthy recruitment phases [25]. Other methods of randomisation varied in success. Sealed envelopes provide a useful and cost-effective method to randomise across the 24 h day; however, triallists must consider where these are placed (i.e. control rooms, remote or individual vehicles) to minimise additional steps to obtain group allocation by telephone that may add time and complexity to the patient care episode [47]. Conversely, an advantage of seeking telephone/remote randomisation is that verification of inclusion or exclusion criteria can be made prior to randomisation. Pseudo-randomisation offers opportunity to test treatments that cannot be blinded or fully randomised. All the MSU studies used an alternating week randomisation schedule since it would be impossible to blind the intervention. Therefore, patients were allocated to the MSU or control according to their week of presentation.

It is clear across all trials in this review that patient outcome and the relationship with prehospital time intervals are of interest to investigate. It is challenging to solely assess the effect of time intervals in the prehospital phase because there is major variation in time interval terminology reported. Future collaboration between triallists on reporting of distinct time phases within prehospital stroke will improve homogeneity for future meta-analysis.

Mechanisms to obtain explicit informed consent in prehospital trials have often been extrapolated from more established medical settings where similar research pressures are seen [48]. Paramedic elicitation of informed consent has been applied in some myocardial infarction prehospital thrombolysis trials but remains problematic given the time-critical nature of the condition, with a requirement to monitor and deliver emergency care [49]. Patients are often too unwell or overwhelmed to provide consent, and next of kin may be too distressed with a multi-page information and consent pack. In stroke, however, due to the varying severity of presenting symptoms, several patients retain capacity and arguably the process of negating, or waiving, assessing capacity for informed consent cannot be overlooked, and this review highlights that each consent method has a place depending on the nature of the research.

Included studies varied in quality, sample size and population. This was somewhat anticipated due to the novelty of research in the prehospital field. There were some methodological weaknesses identified but these relate to issues unique to the multicentre research in emergency settings and as such, four trials set secondary outcomes to assess the feasibility of ambulance-based research. Indeed, all conclude that prehospital research is feasible, but the unique ambulance-based setting presents challenges that warrant further exploration, especially as the paramedic profession continues to cultivate its research capabilities. Ensuring simplicity in future trial designs will undoubtedly encourage participation, promote interest and protocol compliance from Paramedics often working alone in chaotic and time-pressured environments.

### Limitations

We limited studies to those involving an element of randomisation whether true or pseudo, e.g. alternate weeks as used in the mobile stroke unit trials. However, we explored the range and scope of interventions tested and purposely remained broad with no specification of time, language or country limitations.

Summarising the literature in this way poses several challenges. First, these conclusions are drawn from a variety of data sources, countries, time periods and patient populations. Skill levels of medical providers on ambulances vary with country, clinical responsibilities and autonomy and experience of prehospital research. Not every study provided detail on the grades of prehospital staff working within each setting. Similarly, the arrangement of wider healthcare systems is too varied limiting the overall ability to draw conclusions. Not all studies report limitations or challenges in conducting research in ambulance services, and there is disparity in reported time intervals making meta-analysis unfeasible. However, this review serves as an initial insight to develop ambulance-based research for all health conditions, not just stroke.

A wider review inclusive of prospective, non-randomised and observational trials may broaden the scope and complement this review with an overview of contemporary developments in ambulance-based stroke research.

## Conclusion

There is heterogeneity of research design and outcomes measured in the existing body of ambulance-based stroke literature. Whilst prehospital research is evolving, randomised activity is clearly feasible and can be undertaken in the prehospital environment with potential developments for stroke across every point of contact with ambulance services without impacting upon timeliness. However, none have yet resulted in a change to ambulance service practice.

New methods need to be developed to help extrapolate intervention complexity and balance cost-effectiveness to increase generalisability across the wider practice setting. Early consultation between ambulance services and triallists, drawing on research of prior challenges and lessons learnt will contribute to understanding research complexity in the prehospital environment and mitigate some of the difficulties reported here.

It can be concluded from this review, in congruence with the literature, that randomised research is taking place to support prompt identification of stroke [11], deliver organised, timely and efficient emergency department bypass towards appropriate facilities, initiation of treatment on-scene and in-transit to definitive care are all essential and play an integral part of effective stroke treatment. Despite the inconsistencies to yet deliver an efficacious prehospital pharmacological therapy, the feasibility of combining elements across the chain of prehospital care and delivering a prehospital intervention without losing time in the prehospital phase of stroke care is clear.

Future work to overcome challenges within this review to harmonise data acquisition and determine key time parameters will allow broader meta-analysis through collaboration with ambulance services, triallists and stroke networks serving to complement the existing literature in ambulance-based stroke research.

### Supplementary information

ESM 1 (DOCX 386 kb)

## Data Availability

The datasets generated during and/or analysed during the current study are available from the corresponding author on reasonable request and a protocol for their use.
