# Supplementary material for: A systematic review of ambulance service-based randomised controlled trials in stroke
Source: Neurol Sci. 2023 Jul 5;44(12):4363–78. doi: 10.1007/s10072-023-06910-w (PMC10641071; doi:10.1007/s10072-023-06910-w)
Supplement: Supplementary file 1 — (DOCX 386 kb) [file 10072_2023_6910_MOESM1_ESM.docx]

**Itemised list of Tables and Figures**

Supplement Table 1 Included trials

Supplement Table 2 Trials reviewed for inclusion, but excluded on full assessment

Supplement Table 3 Risk of Bias

Supplement Table 4 Time Intervals

Supplement Table 5 Detailed information on included studies and characteristics of ambulance-based research

Supplement Figure 1 Time interval: onset to call/alarm forest plot

Supplement Figure 2 Time interval: call/alarm to arrival at scene forest plot

Supplement Figure 3 Time interval: onset of symptoms to treatment forest plot

Supplement Figure 4 Time interval: time of arrival at scene to treatment forest plot

Supplement Figure 5 Time interval: time of arrival at scene to arrival at hospital forest plot

**Search Strategy**

***Medline Search Strategy***

This search strategy employs the Cochrane Highly Sensitive Search Strategy for identifying randomised trials in MEDLINE: sensitivity-maximising (2008 revision).

*(Adjusted for EMBASE, Web of Science and CENTRAL)*

1. exp stroke/
2. stroke/
3. cerebrovascular accident/
4. 1 or 2 or 3
5. Ambulances/
6. Emergency Medical Services/
7. Emergency Medical Technicians/
8. Paramedics/
9. 5 or 6 or 7 or 8
10. 4 and 9
11. randomi?ed controlled trial.pt.
12. controlled clinical trial.pt.
13. randomi?ed.ab.
14. placebo.ab.
15. clinical trials as topic.sh.
16. randomly.ab.
17. trial.ti.
18. 11 or 12 or 13 or 14 or 15 or 16 or 17
19. exp animals/ not humans
20. 18 not 19
21. 4 and 9 and 20

**Supplement Table 1 Included Trials**

| **Study** | **Year** | **Location** | **Study Period (months)** | **N** | **Design** | **Randomisation** | **Prehospital personnel** | **Primary Outcome and Key Findings** |
| --- | --- | --- | --- | --- | --- | --- | --- | --- |
| De Luca [19] | 2009 | Italy | - | 4895 | Cluster RCT | Cluster level randomization by geographical area | Ambulance Drivers  Physicians  Nurses | Proportion of patients referred to stroke unit  Positive CT for IS within 6 hours:  Power reduced as some symptom onset data missing, one large region withdrew. |
| Nurmi [20] | 2011 | Finland | - | 61 | Prospective, randomised intervention | Sealed envelopes opened in ambulance | Paramedics | Plasma glucose concentration:  *Significant reduction of plasma glucose with IV insulin during the prehospital phase.* |
| Berglund [21] | 2012 | Sweden | - | 942 | Randomised controlled trial | Sealed envelopes in control room | Nurses in control  Nurses in ambulances | 1. Unproportional interference with other ambulance transports: *no obstruction noted* 2. Increase in patients arriving at SU within 6 hours from symptom onset: *shorter time reported from call to arrival at SU with higher priority throughout (P=0.423)* 3. Higher rate of thrombolysis: *significant increase(P=<0.001) patients with higher priority twice as likely to receive thrombolysis in the intervention group*   High mimic rate n=446 (47%) |
| MSU [22] | 2012 | Germany | 32 (Nov 2008 – Jul 2011) | 100 | Randomised single centre-controlled trial | Week-wise strategy | Paramedic dispatcher  On MSU: Paramedic, stroke physician, neuroradiologist | Time from alarm to therapy decision  *Substantial reduction in median time from alarm to therapy decision: 35 min [31-39] min vs 76 [63, 94] min.* |
| Hougaard [23] | 2013 | Denmark | 19  (Jun 2009 – Jan 2011) | 443 | Single centre, open-label, outcome observer blinded randomised study | Telephone randomization with physician | Paramedic | Penumbral salvage  *No significant difference in penumbral salvage, final infarct size and infract growth between intervention and control* |
| RIGHT Pilot [24] | 2013 | UK | 23 (Feb 2010 – Dec 2011) | 41 | Paramedic-delivered, ambulance-based single-city prospective single-blind RCT | Sequenced envelopes stored on ambulance vehicles | Paramedic | Reduction of systolic BP at 2 hours:  *P0.030*  Feasible for Paramedics to undertake randomisation |
| PIL-FAST Pilot [25] | 2014 | UK | 14  (Oct 2010 – Dec 2011) | 14 | Double-blind pilot RCT | Individual Paramedic packs | Paramedic | Prehospital enrolment of 4 patients per month, *not met* |
| PHANTOM-S [26] | 2014 | Germany | 21 (May 2011 – Jan 2013) | 6182 | Randomised week, open label clinical trial. | Week-wise randomization intervention/control | Neuroradiologist, paramedic, radiology technician | Alarm-to-needle time:  *Reduction of 15 minutes, p<0.001* |
| Malekzadeh [27] | 2015 | Iran | - | 246 | Quasi-Empirical | Incoming calls randomly answered by trained or non-trained nurses | Telephone triage nurses | Final diagnosis of stroke  *12% Increased accuracy using CPSS vs standard guidelines, OR1.14. Some under triage noted. Small increase in time to dispatch and time to hospital noted in the intervention group.* |
| FAST-Mag [28] | 2015 | USA | 96 (Jan 2005 – Dec 2012) | 1700 | 3-way multicentre randomised double blind, placebo controlled, pivotal RCT | Single-kit stocked on ambulance vehicle | Paramedic | mRS Day-90: *no significant shift in mRS p=0.28* |
| RIGHT-2 [29] | 2019 | UK | 31 (Sep 2015-May2018) | 1149 | Paramedic-delivered, multicentre, randomized controlled trial | Treatment packs 1:1 randomised intervention/sham, signed out at shift start | Paramedic | mRS Day-90:  *no overall improvement in functional outcome at day-9, p0.69* |
| Larsson [30] | 2019 | Sweden | 60 (May 2013 – May 2018) | 19 | Randomised controlled trial | Open-label treatment, 1:1 patient randomisation | Registered prehospital nurse | Reduction on plasma glucose with 2.0mmol/l 4 hours after randomisation  *Trial stopped due to low recruitment. Safe to be administered, no significant difference noted* |
| Helwig [31] | 2019 | Germany | 30 (Jun 2015-Nov 2017) | 116 | Randomised multi-centre | Week-wise strategy | Paramedic, stroke physician, radiologist | Accurate triage of large vessel occlusion or ICH:  *OPM group: 69.8% accuracy of diagnosis using LAMS*  *MSU group: 100% accuracy,*  *Value in employing both strategies* |
| PASTA [32] | 2020 | UK | 40 (Jul 2015-Dec 2018) | 1214 | Pragmatic, multicentre, cluster RCT | Station clusters with all paramedics at individual stations involved in a pre-specified arm:  PASTA or Standard Care | 1 or 2 paramedics, CT Technologisy, critical care nusrse | Proportion of patients receiving thrombolysis |
| BEST-MSU [33] | 2021 | USA | 72 (Aug 2014 – Aug 2020) | 1515 | Pseudorandomised - alternating week, cluster-controlled trial of a mobile stroke unit | Week-wise strategy |  | mRS at Day-90 of 0 or 1 = 55% in MSU group vs 44% in EMS.  t-PA from onset MSU =72 minutes  t-PA from onset EMS = 108 minutes. |

**Supplement Table 2 Trials reviewed for inclusion, but excluded on full assessment**

| **Study** | **Year** | **Location** | **N** | **Design** | **Rationale for Exclusion** |
| --- | --- | --- | --- | --- | --- |
| Crocco | 2003 | USA | 222 | Prospective trial | Non-randomised |
| FAST-Mag Pilot | 2004 | USA | 20 | Non-randomised, open label clinical trial | Non-randomised method of allocation. |
| Wang | 2004 | USA | 14 | Randomised, controlled-trial | Intervention not applied in the ambulance |
| Leira | 2009 | USA | 100 | Randomised controlled trial | Prior assessment by rural ED physician requesting inter-hospital helicopter transfer to tertiary centre. |
| Muller-Nordhorn | 2009 | Germany | 75,720 | Cluster-randomised trial | Intervention not applied in the ambulance; population based educational letter |
| Berglund | 2014 | Sweden | 942 | Descriptive study of patient populations | Non-randomised |
| PreSSUB II | 2015 | Belgium | 140 | Randomised, open-blinded, end-point single-centre trial | Protocol paper – telemedicine vs. standard care in prehospital stroke |
| Sanossian | 2015 | USA | 863 | Consecutive subject enrolment | Non-randomised method of allocation |
| Shkirkova | 2017 | USA | 572 | Sub-study of FAST-Mag | Sub-study of main FAST-Mag trial |
| Bowry | 2018 | USA | 50 | Consecutive subject enrolment | Not randomised controlled trial |
| Lin | 2018 | USA | 248 | Prospective data collection | Non-randomised |
| Yamal | 2018 | USA | - | Phase III, multicentre, prospective cluster-randomized | Protocol paper |
| Yang | 2019 | China | - |  | Meeting Abstract |
| Koka | 2020 | Switzerland | 39 | Randomised controlled trial | Intervention not applied in the ambulance – NIHSS training of Paramedics, not evaluated in patient setting |
| Yamal | 2021 | USA | 926 | Prospective multicentre cohort study with randomized deployment weeks and blinded assessment of both trial entry and clinical outcomes | Study ongoing |

**Supplement Table 3 Risk of Bias Assessment**

Risk of bias was assessed in accordance with the Cochrane collaboration’s tool for assessing bias in randomised trials. [18]

| **Study** | **Random sequence generation (selection bias)** | **Allocation concealment (selection bias)** | **Blinding of participants and personnel (performance bias)** | **Blinding of outcome assessment (detection bias)** | **Incomplete outcome data (attrition bias)** | **Selective reporting (reporting bias)** | **Other bias** |
| --- | --- | --- | --- | --- | --- | --- | --- |
| De Luca [19] | Low | Low | Low | Unclear | High | Low | Low |
| Nurmi [20] | Low | Low | High | Low | Unclear | Low | Low |
| Berglund [21] | Unclear | Low | Unclear | Unclear | Unclear | Low | Low |
| MSU [22] | Low | Low | Low | Unclear | Low | Low | Low |
| Hougaard [23] | Low | Low | Low | Low | High | Low | Low |
| RIGHT Pilot [24] | Low | Low | Unclear | Low | Low | Low | Low |
| PIL-FAST Pilot [25] | Low | Low | Low | Unclear | Low | Low | Low |
| PHANTOM-S [26] | Low | Low | Low | Unclear | Low | Low | Low |
| Malekzadeh [27] | Unclear | Unclear | Unclear | Unclear | Low | Low | Low |
| FAST-Mag [28] | Low | Low | Low | Unclear | Low | Low | Low |
| RIGHT-2 [29] | Low | Low | Low | Low | Low | Low | Low |
| Larsson [30] | Low | Low | Low | Unclear | Low | Low | Low |
| Helwig [31] | Low | Low | Low | Unclear | Low | Low | Low |
| PASTA [32] | Low | Low | Low | Low | Low | Low | Low |
| BEST-MSU [33] | Low | Low | Low | Low | Low | Low | Low |

**Supplement Table 4 Time intervals**

| **Study** | De Luca [19] | Nurmi [20] | Berglund [21] | MSU [22] | PIL-FAST[25] | RIGHT [24] | PHANTOM-S [26] | Malekzadeh [27] | FAST-Mag [28] | RIGHT-2 [29] | Helwig [31] | PASTA [32] | BEST-MSU [33] |
| --- | --- | --- | --- | --- | --- | --- | --- | --- | --- | --- | --- | --- | --- |
| **N** | 4895 | 23 | 942 | 100 | 14 | 41 | *6182* | 246 | 1700 | 1149 | 116 | 1214 | 1047 |
| **Onset to call** |  |  |  |  |  |  |  |  |  | 19 [5, 64] | I= 63 (89) C= 83 (99) | I= 26 [9, 67] C= 32 [12, 76] | MSU= 23 [8, 52]  EMS= 22 [11, 60] |
| **Call to dispatch** |  |  | I= 5 [4, 7] C= 8 [5, 14] |  |  |  |  |  |  | 3 [1, 7] |  |  |  |
| **Alarm/ Call to arrival at Scene** |  |  |  | I= 12 [9, 16] C= 8 [6, 11] |  |  |  | I= 18.2±5.8 C= 17.2±5.9 |  | 13 [8, 22] |  | I= 22 [14, 36] C= 20 [14,34] | MSU= 9 [6, 13]  EMS= 9 [6, 13] |
| **OTR** |  |  |  |  | 70 [40, 89] | 55 [45, 120] |  |  |  | 71 [45, 116] |  |  |  |
| **OTT** |  | (IV) 57 (±22) (SC) 56 (±27) |  | ***Onset to therapy decision*** *I= 56 [43, 103]*  *C= 104 [80, 156]* ***Onset to thrombolysis*** *I=72 [53, 108]*  *C=153 [136, 198]* |  |  |  |  | 45 [35, 62] I= 45 [35, 60] C= 46 [36, 62] | 72 [45, 116] |  |  | MSU= 72 [55,105]  EMS= 108 [84, 147] |
| **Alarm to treatment** |  |  |  | **Alarm to therapy decision**  I= 35 [31, 39]  C= 76 [63-94] |  |  | STEMO care 51.8 (49.0-54.6) 48 [39, 56] STEMO Weeks 61.4 (58.7-64.0) 55 [44, 75] Control weeks 76.3 (73.2-79.3) 72 [62, 85] |  |  | 43 [32, 57] |  |  | I 46, [39, 55]  C= 78 [66, 93] |
| **Arrival at scene to treatment** |  |  |  |  | 25 [17, 28] |  |  |  | 23 [18, 27] | 22 [15, 31] |  |  |  |
| **Total Time on Scene** |  | (IV) 29 [22, 38] (SC) 20 [17, 23] Control 22 [19, 30] | I= 14 [10, 18] C = 13 [9, 18] | - |  |  |  |  |  | 33 [26, 46] |  | I= 24 [15,34] C= 22 [14, 31] |  |
| **Arrive Scene to arrival at hospital** |  |  |  |  | 38 [32, 42] (32 mins for routine admissions) |  |  |  | 33 [27, 39] I= 32 [27, 39] C= 33 [27, 39] | 50 [40, 64] |  | Scene to treatment I= 90 [72, 114] C= 86 [68, 107] | MSU= 55 [47, 62]  EMS= 27 [21, 33] |
| **Leave Scene to arrive hospital** |  |  |  |  |  |  |  |  |  | 15 [10, 23] |  | I= 14 [10, 20] C= 14 [9, 20] |  |
| **Alarm to Hospital** |  |  |  |  |  |  |  | I= 43.9±10.8 C= 41.7±11.8 |  | 97 [71, 114] |  | *Onset to needle I= 146 [110, 194] C= 137 [110, 190]* |  |
| **Call to Needle / treatment** |  |  |  |  |  |  |  |  |  |  | I= 84.9 (30.2) C=50.1 (10.1) | I= 48.5 [35, 75] C= 48.5 [36, 65] | MSU= 46 [39, 55]  EMS= 78 [66, 93] |
| **Call to EVT** |  |  |  |  |  |  |  |  |  |  |  |  | MSU= 141 [116, 171]  EMS= 132 [114, 160] |
| **Door to needle** |  |  |  |  |  | 67 [53, 83 N=10] | C = 36 minutes |  |  |  |  |  | EMS = 40 [31,51] |
| **Door to EVT** |  |  |  |  |  |  |  |  |  |  |  |  | MSU= 76 [53, 105]  EMS= 94 [72, 124] |
| **Last known well time to EVT** |  |  |  |  |  |  |  |  |  |  |  |  | MSU= 166 [131-160]  EMS= 163 [134-209] |
| **Dispatch to hospital** | EMS Intervention referral to SU= 29.2(12.1) Referral other hosp= 32.6 (15.9) Control Referral to SU= 32.2 (9.2) Referral to other hosp= 35.9 (14.0)  ER Intervention referral to SU= 180 (162) Referral to other hosp 318 (240)  Control referral to SU= 216 (210) Referral to other hosp 288 (240) |  |  |  |  |  |  |  |  |  |  |  |  |
| **Hospital arrival to Paramedic clear** |  |  |  |  |  |  |  |  |  |  |  | I= 36 [27, 50] C= 29 [20, 38] |  |
| **Call to Paramedic Clear** |  |  |  |  |  |  |  |  |  |  |  | I= 102 [85, 123] C=90 [76, 110] |  |

Hougaard[26] and Larsson[31] did not report timings. BEST-MSU[20] report timings for patients eligible for t-PA n=1047.

Data are: Time in minutes, median [Inter quartile range], mean (SD).

Abbreviations: AIS, Acute Ischaemic Stroke; OTR: Onset to treatment; OTT: Onset to treatment STEMO, STroke Emergency Mobile, EVT: endovascular thrombectomy

**Supplement Table 5 Detailed information on included studies and characteristics of ambulance-based research**

| **Study** |  |
| --- | --- |
| De Luca [19] | - Cluster RCT involving 52 ambulances from 29 EMS Stations, in 20 randomisation units. Numbers of staff participating, and skill levels not reported. - Resource intensive to co-ordinate and implement training, organisation. - Significant coordination required to overcome differences in regional management, socio-demographic factors and road networks. - Despite efforts, low patient referral seen. - Long transfer distances to the Stroke Unit and unavailability of ambulances to transport led to the withdrawal of a participating region. - Little change was reported in EMS times from dispatch to arrival stroke unit in the intervention group instead of closest hospital – but given the increased distance, the authors attribute the minimal difference to the effect of training in CPSS and stroke care. - EMS personnel omitted to record symptom onset information in some patients (n not reported), a requirement for trial eligibility |
| Nurmi [20] | - 5 EMS ambulances took part, conveyance of patients to one hospital - Authors report early identification and fast transportation is of utmost importance. - Consent, study related procedures and administration of IMP contributed a delay of 7 minutes. |
| Berglund [21] | - Training delivered to control centre staff and EMS staff – n not reported. - EMS Crews were able to include patients on scene where stroke not recognised by EMCC. |
| MSU [22] | - Geographical considerations meant some patients were unable to be scanned due to location on hillside (steep streets, n=2) or overweight (n=2) |
| Hougaard [23] | - No reported numbers of ambulance-staff participating or skill level - Randomisation imbalance due to misunderstanding with initial consent recording – patients randomised to control group did provide final written consent. Data lost to follow up, number of patients lost unknown. - No other ambulance-based references noted. - Methodology paper reported intended sample size 120, actual reported sample 443. |
| PIL-FAST Pilot [25] | - 76 Paramedics trained, lower recruitment rate than anticipated. - ¼ study medication was lost at handover between ambulance and hospital - Four patients lost due to incorrect onset judgment time by Paramedics - could be improved with training but reflects challenge of recruiting to trials at the scene. |
| RIGHT Pilot [24] | - Lower recruitment rate than anticipated n=41, 1.8 patients per month. - 32 patients (78%) randomised within 120 minutes of symptom onset - 19 patients enrolled outside working hours - Recruitment limited to working hours only - 78 Paramedics trained from 12 ambulance stations. 23 of whom randomised patients - Training was face-to-face on ambulance stations - Paper reports ‘time from Paramedic review to leaving scene did not differ’ - unclear what was defined as Paramedic review? |
| PHANTOM-S [26] | - Catchment area limited to 16-minute radius, within time limits of 07:00-23:00 - 1x vehicle - Specialist staff required to operate the vehicle, delivery scan and thrombolysis: Neurologist, paramedic and radiology technician. - Monocentric: is it cost effective? |
| Malekzadeh [27] | - Unknown number of nurse call-handler participated. - CPSS required longer call duration to assess, increasing dispatch and response times. However results demonstrate an increase in accuracy of anterior-circulation stroke diagnosis and suggest a reduction in over-triage. |
| FAST-Mag [28] | - 3000 Paramedics, 315 Paramedic ambulances - ¾ of patients received the intervention within 60 minutes. - Two step enrolment process – Paramedic LAPSS then physician LAMS over the telephone, reduction of mimic enrolment to 3.9% - Pre-study arrive at scene to hospital time 34 minutes. Study intervention did not delay time from arrival at scene to arrival at hospital 34 minutes. - Methodology paper reports that Paramedics attended a 1-2 hour training lecture on stroke pathophysiology and assessment. In depth training, that may not be reflective. 8-year duration required significant retraining - Pre-stocking randomisation technique to minimise bias |
| RIGHT-2 [29] | - 1492 Paramedics trained from 8 participating ambulance services, 516 randomised at least one patient. These Paramedics were based at 186 ambulance stations. - 54 primary stroke centres participating - Recruitment initially restricted to working hours but extended to 24 hours 7 days. - Paramedics unmasked to treatment following randomization, participants remained blinded. |
| Larsson [30] | - Low recruitment stopped trial; protocol amended to broaden blood sugar range to increase recruitment. - Limited by written informed patient consent only - Prolonged length of study - Training of nurses, 90 investigators, move stations, services therefore significant retraining required. - Maintaining drug temperatures stocked on the vehicle |
| Helwig [31] | - Two ambulance stations selected, acknowledged boundary of area 16-minute travel time from station to scene - Distance reported from Station to Scene: I = 4.0km (2.3) C= 4.1km (3.5) - State-wide structured training sessions required every three months for EMS personnel in practice and 6 months for personnel in the dispatch centre - Consent was confirmed by study physician on arrival at the receiving hospital. |
| PASTA[32] | - 227 data collection forms out of 500 located - 242 PASTA trained Paramedics and 335 standard care paramedics recruited average 2.0 patients per paramedic. - Cluster randomisation by ambulance station chosen methodology to remove complication at scene - Patients confirmed enrolment after confirmation of stroke diagnosis by local investigator |
| BEST-MSU [33] | - 72-month recruitment phase - Written informed consent from all patients or their representative - Seven recruitment cities with varying hours: Mon-Fri recruitment 0800-1700 Houston / Mon-Fri 0800-2000 at 4x sites / Thurs-Tues 24 hour at x2 sites / Mon-Fri 0700-1900 x1 site - Low recruitment in six sites, main Houston trial centre city achieved highest recruitment |

**Supplement Figures**

**Supplement Fig. 1 Time interval: onset to call/alarm forest plot**


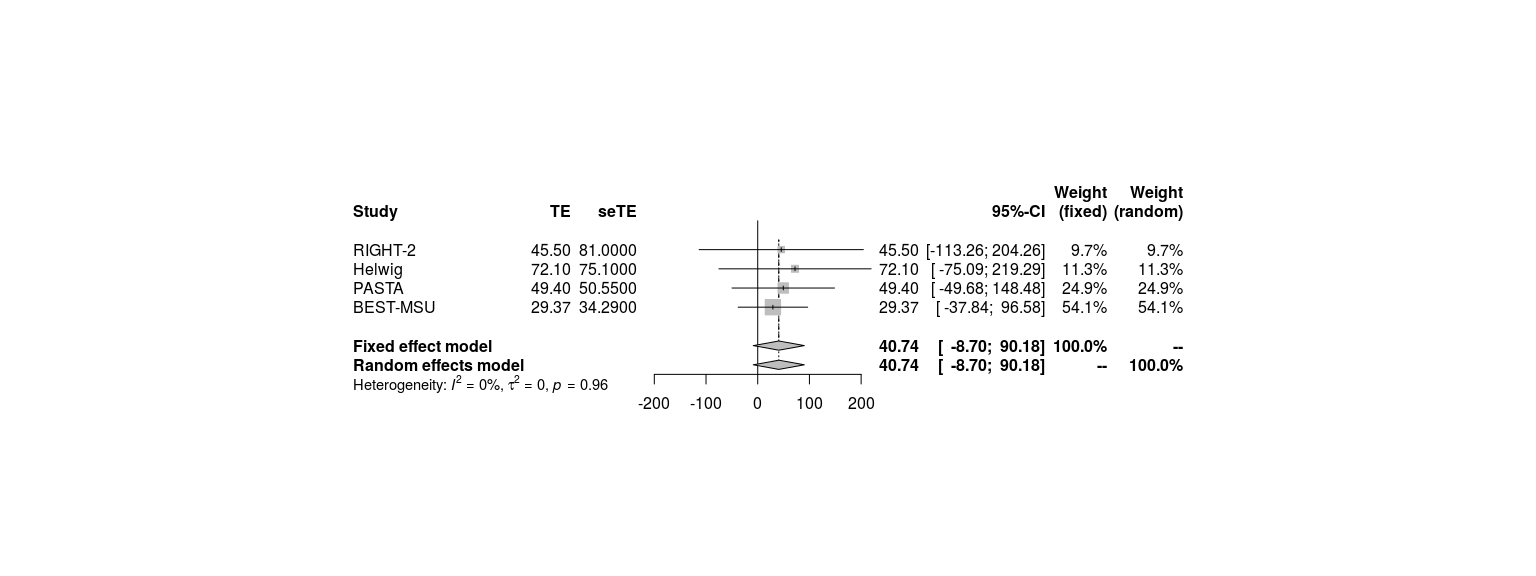


**Supplement Fig. 2 Time interval: call/alarm to arrival at scene forest plot**


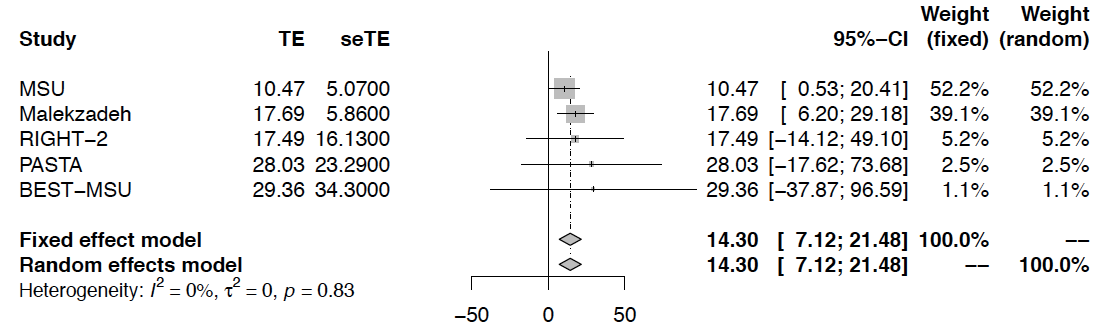


**Supplement Fig. 3 Time interval: onset of symptoms to treatment forest plot**

**
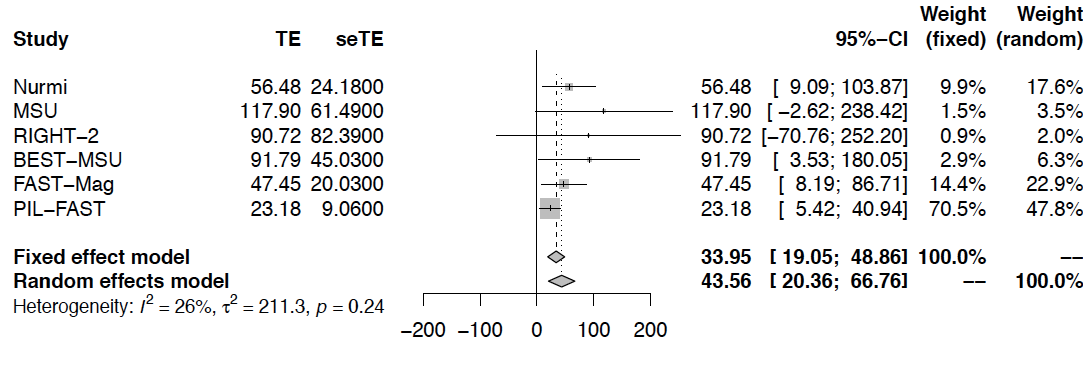
**

**Supplement Fig. 4 Time interval: time of arrival at scene to treatment forest plot**


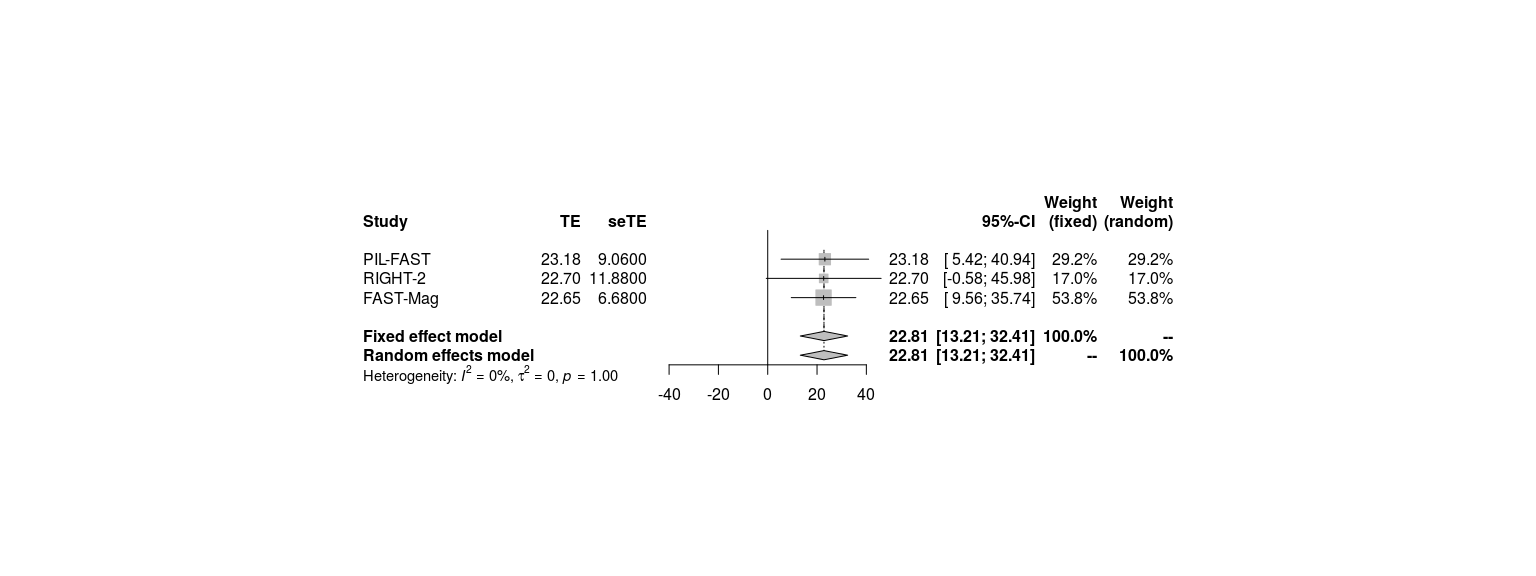


**Supplement Fig. 5 Time interval: time of arrival at scene to arrival at hospital forest plot**

**
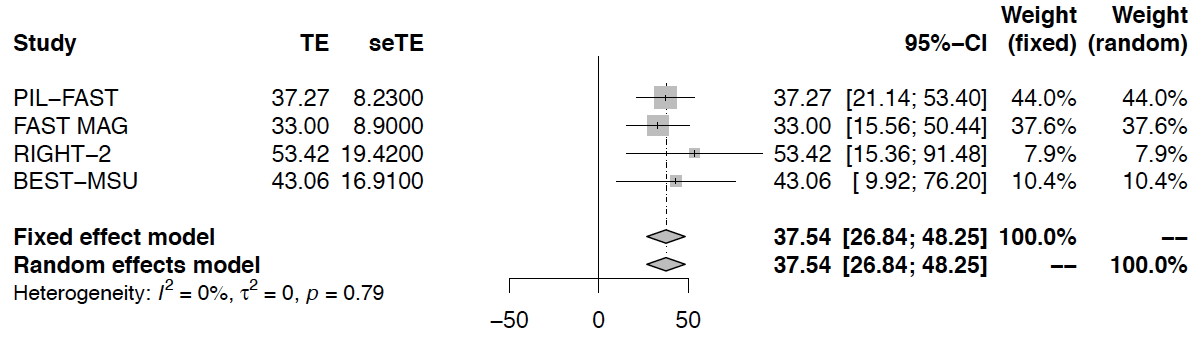
**
